# Drawing parallels in human–other interactions: a trans-disciplinary approach to developing human–robot interaction methodologies

**DOI:** 10.1098/rstb.2018.0433

**Published:** 2019-03-11

**Authors:** Emily C. Collins

**Affiliations:** Department of Computer Science, The University of Liverpool, Liverpool, UK

**Keywords:** human–robot interaction methodology, touch, attachment, caring, animal-assisted therapy, trans-disciplinary

## Abstract

This opinion paper discusses how human–robot interaction (HRI) methodologies can be robustly developed by drawing on insights from fields outside of HRI that explore human–other interactions. The paper presents a framework that draws parallels between HRIs, and human–human, human–animal and human–object interaction literature, by considering the morphology and use of a robot to aid the development of robust HRI methodologies. The paper then briefly presents some novel empirical work as proof of concept to exemplify how the framework can help researchers define the mechanism of effect taking place within specific HRIs. The empirical work draws on known mechanisms of effect in animal-assisted therapy, and behavioural observations of touch patterns and their relation to individual differences in caring and attachment styles, and details how this trans-disciplinary approach to HRI methodology development was used to explore how an interaction with an animal-like robot was impacting a user. In doing so, this opinion piece outlines how useful objective, psychological measures of social cognition can be for deepening our understanding of HRI, and developing richer HRI methodologies, which take us away from questions that simply ask ‘Is this a good robot?’, and closer towards questions that ask ‘What mechanism of effect is occurring here, through which effective HRI is being performed?’ This paper further proposes that in using trans-disciplinary methodologies, experimental HRI can also be used to study human social cognition in and of itself.

This article is part of the theme issue ‘From social brains to social robots: applying neurocognitive insights to human–robot interaction’.

## 1. Introduction

Human–robot interaction (HRI) is a highly trans-disciplinary field. As such, HRI has faced multiple challenges in asking how to create a distinct experimental methodology and theoretical practice to unify the many disciplines it draws upon. However, this is changing. The year 2018 saw the advent of an official *HRI ACM Transactions* journal, and special editions, such as this one, stand in recognition of HRI as an area of research in its own right. The need for HRI is growing. Industry 4.0, and the global trend towards ever smarter factories calling for advancements in co-botics, is upon us [1], while the growth of social robots, for use in both domestic and work environments, continues to raise questions of what constitutes a social agent worthy of human attention. Despite this need and growth, the field of HRI continues to suffer from a lack of precision and clarity in the work it produces. Kompatsiari *et al.* [2] note that measures selected to evaluate HRI often consist in self-assessments and behavioural methods, but lack experimental control, or statistical power in the sample. If a robot appears to be good at something the question should always be asked, but why is this the case? It is not enough to simply see a positive or negative effect of interacting with a robot. A mechanism for such an effect must also be sought. How can interdisciplinary researchers be consistent with the methods they choose, without established theoretical foundations upon which to base such tooling selection decisions?

This opinion paper will discuss how HRI methodologies can be robustly developed by drawing on insights from fields outside of HRI that explore human–other interactions. It will begin by defining HRI as a system, comprising three aspects that exist within a given environment. It will then outline an abstract human–other agent interaction framework to explain how drawing parallels between HRIs and human–human, human–animal and human–object interactions, by considering the morphology and use of a robot, can aid the development of robust HRI methodologies. This opinion paper will then briefly present some novel empirical work as proof of concept for the framework. In drawing on known mechanisms of effect in animal-assisted therapy (AAT), and social-cognitive methods related to behavioural observations of touch patterns and individual differences in caring and attachment styles, this example will demonstrate how methods can be co-opted from fields outside of HRI to explore the mechanisms of effect occurring within a human–biomimetic robot interaction, given the robot's desired use within the analogous robot-assisted therapy (RAT) field. This will highlight the importance of understanding the role of the human, the morphology and purpose of the robot, and the desired outcome of the interaction, within a given context, for optional HRI theory and robust experiment creation.

By drawing on fields in which other human interactions are studied, and exploring what a robot is in light of the other agents considered in human–interaction dyads, the field of HRI not only forms a strong theoretical foundation that lends itself to bringing reproducibility and validity to its experiments, but further can result in a deepened understanding of how humans interact with novel agents in the world (i.e. robots, in all their many forms), reciprocally benefiting those fields from which HRI methods have been drawn.

## 2. Human–robot interaction is a system: modelling interactions by drawing parallels

HRI is a system comprising three parts: a human element, a robot element and the interaction of these two agents therein. To understand what is happening within that system each aspect should be clearly defined. Furthermore, the context in which that HRI is taking place must also be clearly understood. To create robust empirical work, an HRI researcher should consider all of these definitions for their given HRI context prior to devising the experiment.

For example, this opinion paper proposes that a robot can be defined by its morphology and its use. A robot is a tool to do something, but once a level of autonomy has been introduced to it, the question of the robot's agency is raised. Bestowing a robot with ‘agency’ will change how the human partner considers the tool: no longer simply as something to be relied upon to produce end goals with, but as a distinct agent with, potentially, its own competing goals. A researcher should ask: What is the robot to be used for? Is it to provide a social partner with a distinct agency or is it to be an advanced tool that will only comply with its user's desires?

Robots are liminal: neither alive nor inanimate, occupying a position on either side of an otherwise clear boundary in being objects, but ones that behave autonomously and, in the case of a social robot, are capable of social interaction [3], or in the case of any robot, are capable of eliciting a social-cognitive response from a human engaging with it, by a robot's nature as an interactive agent in the world. Anecdotally, there are numerous reports of humans displaying behaviour towards their robots akin to what one might witness from an individual towards their pet dog [4].

The human element is not universal either. For each HRI scenario, a responsible researcher should ask: Who is the human in *this particular* HRI system? What are they doing in this context? What do they want from the interaction? How will they perceive the robot, given the answers to these questions? In addressing these questions, it becomes clearer how important, and useful, psychological measures of social cognition can be for deepening our understanding of HRI and developing richer HRI methodologies. Humans are a gregarious species, with an intelligence rooted in sociality [5]. This social motivation underlies a psychological disposition to anthropomorphize the environment, that is, to ascribe human form or attributes to another, including animals, plants and objects. Epley *et al.* [6] argue that anthropomorphism serves the purpose of reducing uncertainty and increasing confidence in predictions, by bestowing upon the anthropomorphized other known quantities of human characteristics and motivations. Non-human agents are unknowns. In the absence of social connections, individuals tend towards using anthropomorphism to create human agents from non-human entities in order to satisfy their own need for social connection [4]. Dautenhahn [7] writes that a human might anthropomorphize a coffee machine, or easily pretend a plush toy horse is alive when playing with a child. The phenomenon of pareidolia reveals the extent to which humans see living form in their environment. Minimal morphology, such as a line and two circles, can be enough for an individual to attribute human characteristics to even the most formless of objects. Thus, an interaction with a robot with a level of autonomy, whether that be a social, industrial or another kind, is capable of drawing upon a human's social cognition.

Given this, although it is clear that robots do not belong in the same class of living things to which humans and animals do, it stands that robots do have capacities for interaction and appearing ‘alive’ far beyond those of inanimate objects. As such, when developing HRI theory and methodology, it is also important to define what sort of interaction the two agents are required to engage in, in order to obtain the desired outcome, while also ensuring that this is done with a clear understanding of the environmental, physical and other contextual factors framing where the interaction is taking place.

Answering these questions gives the researcher a way to begin to develop a theoretical foundation upon which to understand the specific HRI being addressed. Given the diversity in morphology and use among robots, one way of approaching this is by drawing from three distinct bodies of human–other interaction literature: the interactions humans have with other humans (from those rooted in attachment bonds, to more general interactions humans have with strangers); the interactions humans have with animals and the interactions humans have with objects.

There have been some attempts by HRI researchers to apply metrics and methodologies from at least human–human interaction (HHI) research in their assessment of robot performance. For example, in the application of understanding of gaze, Staudte & Crocker [8] were the first HRI researchers to apply HHI psychological findings regarding gaze to an HRI experimental design. In HHIs, referential gaze has been shown to be instrumental in the planning process of utterance production [9]. Inspired by this and other works in the area of HHI gaze, Staudte & Crocker employed gaze methodologies to demonstrate that humans react to robot speech and gaze in the same way they would to another human.

HHI proxemics, that is the study of how humans use and manipulate distances, has also been applied to HRI research. Walters *et al.* [10] used the Human–Human Personal Space Zone metric [11], based on earlier proxemics work by Hall *et al.* [12], to inform their proposed human–robot proxemics framework by way of comparison. Sardar *et al.* [13] used HHI proxemic's metrics provided by Hall [14] to demonstrate that participants provide less compensatory behaviour when having their personal space invaded by a human compared with a humanoid robot.

Individual differences are also important to understanding how social cognition differs among populations. However, research focused on the application of individual differences to HRI is scarce. A study by Syrdal *et al.* [15] explored the relationship between individuals' personality and robot direction preference. Personality was measured via the Big Five Model [16], a domain scale that records emotional stability, extraversion, agreeableness, conscientiousness and intellect. No consistent significant results were obtained to show a relationship between the measured personality traits and preferred approach direction, and the authors, in particular, note that the effect sizes were too small to be significant in the sample size (*n* = 42).

With respect to methodology, however, it should be noted that without established theoretical foundations upon which to base decisions, the highly interdisciplinary field of HRI is often inconsistent with its selection of appropriate tooling. Consequently, results may be prejudiced because tooling was selected on the basis of its expected outcome, and not on the basis of whether it was entirely suited to the task or not. A researcher may well select an instrument from, for example, attachment theory to assess a robot interaction based on what is hoped to be found, and not on what the constraints of the agents involved will theoretically allow. For instance, attachment theory can be useful for exploring HRI, as will be discussed in §3 of this opinion paper, and the general concept of attachment and some of its related measures have been extended for use outside the HHI field by researchers exploring human–animal and human–object relationships. In these cases, the fact that there is a non-human half of a dyad being discussed is taken into consideration. Similarly, any application of attachment theory for the purpose of better understanding HRI should consider the fact that robots are not humans, and subsequent results obtained from studies that include elements of attachment theory as part of their design should be interpreted accordingly. This can be done by, for example, comparing the application of attachment theory in the existing literature on human–object, as well as human–human, interactions in order to form a theoretical basis on which to make predictions regarding how attachment theory might best be applied to understand HRIs. Furthermore, this should be done in light of the particular role of any given robot: its purpose and an example use case, as well as the specific experimental set-up in which it is being assessed. In summary, tooling use requires theoretical and practical context to be efficient. A theoretical foundation that considers the human and their general relationships as central is one way to provide a conceptual framework upon which the description and analysis of HRI can be laid.

It should also be noted that aside from considering a robot as a mere tool, the concept of a robot as a social agent that can be emotionally bonded with has tended to occupy one of two extremes in recent HRI research. At one end is the idea of robots as objects towards which attachment and love should be directed, with the possibility that such human–robot love could be bi-directional [17], and at the other sits the view that robots should be seen as mere objects of ownership, things with the potential to facilitate human–human social bonds but not to be bonded with [18]. However, as robot capabilities advance, they could be developed across the full spectrum of human–other interactions and bonds.

**Figure 1.**
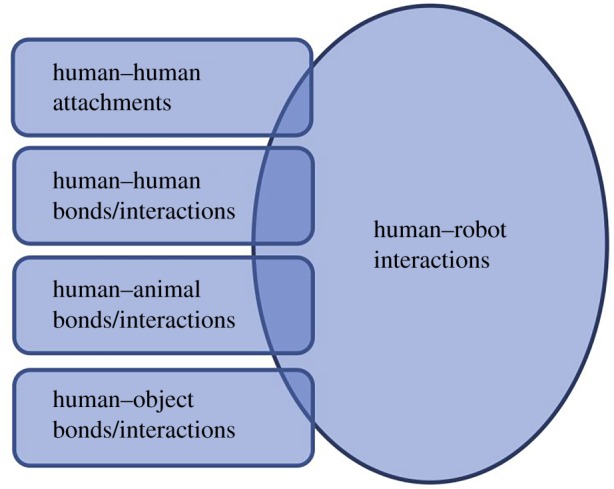
Framework for conceptualizing HRI by comparing morphology and use of robot against other agents/objects with which humans interact. Note that the terms ‘bond’ and ‘interaction’ are grouped together to reflect the broad nature of human interactions with other agents and objects in the world. A ‘bond’ is here defined as an emotional connection, while an ‘interaction’ is any connection with another agent or object in the world. The HRI label does not include the term ‘bond’ as it is representative of the term HRI. However, this does not preclude the possibility that a human could have an emotional connection, and thus a ‘bond’, with a robot. (Online version in colour.)

Existing literature on human–other interactions, including attachments, bonds and relationships, provides established theories and methodologies for exploring how individual differences impact upon a person's interactions with the world. As such, given the social potential of all robots, HRIs can be conceptualized as existing somewhere within this human–other relational space. This opinion paper, therefore, proposes that it is possible to draw parallels with other human interactions to provide a consistent framework with which to describe and analyse HRIs. This framework considers the areas of overlap between a particular robot and its living, or static-object, counterpart, such that for any given robot, its exact location within this space will depend to a large extent on the context in which it is used, as well as its morphology. A humanoid robot designed for social interaction should be explored from an HHI perspective, while a biomimetic robot designed for social facilitation therapy from a human–animal interaction (HAI) one. Such a clear format lends itself to the production of more precise HRI experiments. Rather than simply appropriating psychological metrics to explore in any particular experiment, this framework offers a solid comparative theory to found metric selection upon (figure 1).

It must be stressed that this conceptualization of HRI is not designed to state equivalence between, for example, HAIs and HRIs. Robots are not human, not animal, not static objects, and as analogous as HHI might be to HRI, direct mapping of metrics from one to the other cannot be expected to provide equivalent results. Rather, the framework presented here is designed to provide an understanding of HRI that allows an experimenter to use analytical tools similar to those that already exist for analysing one category of interaction, to analyse the other. In doing so, a researcher can take advantage of the similarities that different interactions have with one another. In short, the comparative approach provides a researcher with a theoretical foundation upon which to make tooling selection, and to aid appropriate analysis, by analysing HRIs in terms of their similarities to other types of existing interactions with other humans, animals and objects (figure 1; see also [19]).

In the following section, this opinion paper will briefly present some novel empirical work as proof of concept, exemplifying how this framework can help researchers select appropriate methodology in order to explore the mechanisms of effect occurring during a specific human–biomimetic robot interaction. The empirical work draws on known mechanisms of effect in AAT, and behavioural observations of touch patterns and their relation to individual differences in caring and attachment styles, and details how this trans-disciplinary approach to HRI methodology development was used to explore how an interaction with an animal-like robot was impacting a user. There is an open question in AAT regarding the extent to which the therapy needs to be tuned to the personality type of the patient, while within RAT the parameters of personality that are important to the successful outcome of an intervention remain unknown. This study is an example of how methodologies applied within social-cognitive psychology, to explore an interaction with a human and an animal in a given context (in this case, social facilitation AAT), can be successfully co-opted in HRI to theoretically ground a study and systematically look for a mechanism of effect.

## 3. Applying the framework

The framework suggests that when selecting methods to conduct a study exploring how an animal-like robot designed for AAT is impacting its user, it is useful to look to methods used in AAT itself to explore how humans interact with therapy animals. Further, the framework, in taking the human as central to all interactions, encourages researchers to consider the importance of individual differences between humans and how they interact with agents in given contexts. Within AAT, mechanisms of effect such as differences in Felt Security (FS) and attachment styles are used to guide how AAT will impact the treatment given to individuals in therapy. Guided by this, it was decided that exploration of a RAT robot's mechanism of effect should employ theory and methods also used to explore mechanisms of effect in AAT, to see whether a RAT robot is working in a similar manner.

### (a) Comparing animal-assisted therapy with biomimetic robot interactions

Animals contribute to their owners' sense of self and well-being, in their need to be cared for and in their ability to display non-judgemental, accepting and attentive behaviour in return for their owners' attention [20]. Animals in general, but in particular companion animals, have been regarded as contributing to human physical and mental health. As such, animals currently play a role in therapy as co-therapist, facilitating a patient or client's existing treatment programme. One form this takes is in being a social focal point resulting in relaxing effects for the human in the short term and improved health benefits in the long term [21]. Animals can be used to help build rapport between the client and the therapist, for example by addressing the initial nervousness felt by a client in a treatment scenario with their enthusiastic greetings.

Extensive medical literature exists confirming a strong link between social support and improved human health and survival [22]. Although AAT is known to positively impact a multitude of conditions, from depressive and anxious symptoms [23], to non-pharmacological pain management [24], the precise mechanisms underlying these effects are subject to debate. However, the benefits derived from supportive social relationships, even those provided by animals, are undoubtable. Any relationship in which a person feels cared for, loved or esteemed is deemed to result in better health and well-being for the individual [21].

The objective of RAT is to create robots with the capacity to act as animal surrogates for individuals who do not have access to animals [25]. RAT has been employed as a successful intervention in areas similar to AAT (e.g. [26]). However, it should be noted that the idea behind RAT is not to replace animals, but to create beneficial opportunities for individuals in situations where AAT is not otherwise possible.

The most widespread application of biomimetically driven RAT has been in the area of elderly care, in particular, dementia treatment programmes that employ animal-like robots. PARO is one of the most active commercial examples of a therapy robot intended to be used in a manner akin to AAT. PARO is an interactive robot, modelled after a baby Canadian harp seal, developed by the Intelligent Systems Research Institute (ISRI). It is marketed as a therapeutic tool for use in nursing home settings, engaging its user with basic capabilities that mimic live animal behaviours. The relationship that develops between a user and the robot is built upon the limited reactions the robot makes to the user's spoken and physical actions [27].

However, what is the mechanism of effect here? How is a RAT robot's performance to be measured? Can the tools used to measure performance in AAT be used equivalently in RAT? To answer these questions, methods from social-cognitive psychology can be employed, to ask what is the pathway of social cognition taking placing during these interactions that results in the positive outcomes we seek?

### (b) Using touch to measure individual differences

FS is an important concept in AAT and is made up of four components: care, love, self-esteem and safety [28]. This can be measured by the Felt Security Scale (FSS; [29]). The FSS was developed as an attachment relationship priming manipulation check for use in HHI studies, although the concept of FS has also been used in HAI studies [30].

Touch is an important mediating effect in AAT, with physical engagement between a human and an animal cited as a mechanism through which positive effects of that interaction are had [31]. While such reported positive effects of physical engagement with an animal via touching behaviours are often anecdotal, or minimally explored in a controlled setting, it remains a canon within AAT that the act of stroking an animal improves patients' feelings of self-esteem and helps patients confined to clinical settings to feel calmer [32]. Research in social neuroscience on the impact of interpersonal touch and its importance to inter-agent affiliation and social bond formation may offer some explanation to such a canon. CT (C tactile) afferents are a type of mammalian, unmyelinated, low-threshold mechanoreceptors, which respond to being touched lightly [33], and play a role in oxytocin release [34], the neuropeptide commonly associated with the communication of emotions and strengthening of social bonds, and point to mechanisms of touch being key to understanding emotional processing in humans.

Given this, the proof-of-concept study to be presented here asked whether the type and amount of touching behaviours with which an individual physically engaged PARO could be correlated against observable changes in FS, given the importance of relaxation to components of FS such as safety and care [35]. This was achieved via the development of a coding scheme that categorized touching behaviours along a detailed continuum of observed behaviours spanning no touch, active touch and intimate touch, producing a set of scores for each participant that conceptualized how they had physically engaged with PARO during an interaction phase.

It was predicted that participants who spent the majority of their robot interaction time physically engaging with PARO in more intimate touching behaviours (for example, stroking PARO while holding it) would have higher pre-interaction FSS scores, and greater FSS change scores from pre- to post-interaction, overall, than individuals who spent the majority of their interaction time physically engaging with PARO with more distant touching behaviours (such as engaging in poking behaviours, or not touching PARO at all). However, if physically engaging PARO with more intimate touching behaviours does lead to greater FSS change scores, then the question arises as to what might drive an individual to physically engage with PARO in a certain way, and furthermore, might there be differences between individuals and their touching behaviours such that certain populations will consistently derive greater benefits from an interaction with PARO than others? To that end, literature exploring individual differences in caring and attachment styles was also co-opted for the study.

In HHI literature, a propensity to physically engage others with intimate behaviours, such as those deemed intimate or caressive, has been linked to individual differences in attachment and caring styles [36]. Attachment style is arranged around an interacting continuum of anxiety and avoidance, to form four attachment styles: secure (low avoidance and low anxiety), preoccupied (low avoidance and high anxiety), dismissive-avoidant (high avoidance and low anxiety) and fearful-avoidant (high avoidance and high anxiety). This is commonly measured with the well-established Experiences in Close Relationships (ECR) measure of adult attachment [37]. Caring orientation is measured with the Caregiving System Scale (CSS; [38]), which scores individual differences in caring hyperactivation and deactivation. In summary, individuals with deactivated caring (or avoidant attachment) styles have a tendency to deactivate emotional responses and may touch animals or others more distally (or not at all), while individuals with hyperactivated caring (or anxious attachment) styles have a tendency to hyperactivate emotional responses and may touch animals and others more intimately.

Therefore, it was also predicted that participants who tended towards hyperactivated caring, or anxious attachment styles, would be more likely to touch PARO more intimately during an interaction phase and would subsequently see greater increases in their FSS score from pre- to post-interaction.

### (c) Example study

Sixty healthy participants (31 female; mean age = 24.42, s.d. = 3.99), with no known physical, auditory or visual impairment, were recruited for the study, which took place in the Sheffield Robotics HRI Laboratory at The University of Sheffield. Upon arrival, participants completed a demographics questionnaire, the 12-item ECR-S [39], the 20-item CSS [40] and the 16-item pre-session FSS [29]. Upon completion, participants were taken to the HRI laboratory and left alone for 5 min with an active PARO, at which time the experimenter returned and asked the participant to complete a second, post-interaction, FSS. The cameras in the HRI laboratory are discrete, and it is not immediately obvious they are there. Attention was not drawn to them by the experimenter. After the study had ended, participants were informed that the session had been recorded.

Video data were coded for measures of physical engagement with PARO. Two categories were created to be coded for alongside one another: Tactile, defined as the hand observed as most engaged in an interaction with PARO, or an observed nose nuzzle that was coded for in this category regardless of hand position because a nose nuzzle represents the most intimate behaviour observed during the interaction sessions, and Position of PARO. These categories were combined to make a 23-item classification system encompassing all observed behaviours (table 1).

**Table 1.** When combined the Tactile and Position of PARO categories make 23 possible actions to be observed during the video-recorded interaction sessions. Held away and held against refer to proximity of PARO to participant's chest.

code	Tactile	Position of PARO
0	no touch	table
1	other touch	table
2	fingertip poke/hold	table
3	whole hand touch	table
4	fingertip stroke	table
5	whole hand stroke	table
6	nuzzle with face	table
7	other touch	held away
8	fingertip poke/hold	held away
9	whole hand touch	held away
10	fingertip stroke	held away
11	whole hand stroke	held away
12	other touch	lap
13	fingertip poke/hold	lap
14	whole hand touch	lap
15	fingertip stroke	lap
16	whole hand stroke	lap
17	other touch	held against
18	fingertip poke/hold	held against
19	whole hand touch	held against
20	fingertip stroke	held against
21	whole hand stroke	held against
22	nuzzle with face	held against

Next, variables were extracted from these coded behaviours to reflective how distal or intimate a participant was with the robot during their interaction session. Three categories were chosen: no touch, active touch and intimate touch.

To decide which of the 23 observable behaviour combinations would belong to each category, values were assigned based on how distal or proximate the participant and robot were to each other. Thus, Tactile behaviour ran from no touch valued at (0), to nuzzle with face valued at (6). While Position of PARO ran: table = (0); held away = (1); lap = (2); and held against = (3). When combined these two values created a number reflecting the observed behaviour along a continuum from no touch/PARO on the table (most distal), to nuzzle/holding PARO against the body (most proximate/intimate). Thus, every participant could be given a value denoting in which of the three categories (no touch, active touch and intimate touch), they spent the majority of their time with PARO.

**Figure 2.**
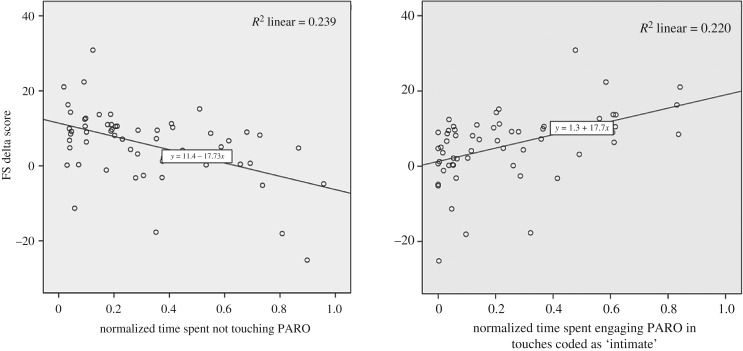
Significant regression equations on FSS change score of: no touch, *F*_1,58_ *=* 18.27, *p* *=* 0.001 and intimate touch, *F*_1,58_ *=* 16.37, *p* *=* 0.001.

To explore whether there was a relationship between how a participant physically engaged with PARO during the interaction period and their FSS change score, simple linear regressions were calculated to predict FSS change score (dependent variable) based on each of the three categories of touching behaviours: no touch, active touch and intimate touch. A significant regression equation was found on FSS change score of no touch, *F*_1,58_ *=* 18.27, *p* *=* 0.001, with an *R*^2^ of 0.239, and on FSS delta score of intimate touch, *F*_1,58_ *=* 16.37, *p* *=* 0.001, with an *R*^2^ of 0.220. These relationships are visualized in scatterplots (with lines of best fit) in figure 2.

However, all other results were not conclusive. No correlations were found between individual differences in caring and attachment styles and how participants physically engaged with PARO in the laboratory. The prediction that individuals who scored highly on hyperactivated caring, or anxious attachment styles, would be more likely to touch PARO more intimately during the interaction phase was not seen. Without a significant connection between individual differences and touch styles, no mediation analysis could be conducted to explore how individuals who were more likely to touch PARO would subsequently see greater benefit from the interaction, as measured by increases in FS. In fact, results revealed direct contradictions to this prediction, for example, some individuals who fell into distinct physical engagement categories (e.g. intimate and no touch), scored equivalently on ECR-S.

The framework encouraged the selection of robust methods used in equivalent HAI settings. By looking at how mechanisms of effect are sought in RAT's comparable area of AAT, the study here presented demonstrates how very specific questions could be asked for this particular HRI scenario, driving the study towards the discovery of a specific explanation of the observed HRI. Although the results were not entirely conclusive, the experience of using an understanding of the socio-cognitive complexities involved in human–other interactions was fruitful. No reason was here found to explain the connection between an interaction with PARO and the subsequent change in participants' FS score, but this observation has driven future research avenues, and poses questions about how human social cognition influences human interactions with biomimetics robots. We are left still asking, why does stroking a biomimetic robot result in changes to a human's feelings of care, love, self-esteem and safety, in a manner similar to those observed when a human strokes a dog?

## 4. Conclusion: trans-disciplinary theories improve human–robot interaction methodologies

The study presented in this paper demonstrated that even after controlling for individual differences in caring and attachment style, time spent with PARO is significantly related to positive increases in FS, such that more time spent engaging PARO in intimate touches leads to greater FSS change scores, while spending more time not touching PARO at all leads to smaller FSS change scores. Thus, not only did engaging with PARO positively affect FS, but how a person engaged with PARO also impacted their outcome FS level, while caring and attachment style could not be used to predict how a person would engage PARO.

The lack of significant impact of individual differences on observed touch behaviours does have parallels in AAT. Here, knowledge of a client's attachment style, though informative for the development of goodness of fit between a client's individual attachment needs and their relationship with their AAT animal, remains advisory and is not prescriptive. This is because identical observed behaviours do not always have equal intent. If Person A scores highly on attachment-anxiety and Person B scores highly on attachment-avoidance, both might still be observed engaging in intimate touching behaviours when interacting with a dog: Person A because they are more prone to physically engage in such a way with other agents in the world and Person B because they do not physically engage so intimately with other humans, and consequently enjoy the company of dogs.

The broad variation in how individuals engaged with PARO, and the lack of impact that the diversity in individual differences was having on those interactions, could be indicative that the value of interacting with PARO in this context is more universal, though given the time constraints in the reported small study this is a bold claim, but one that merits future research. However, if a therapeutic robot can have an equally positive effect across a range of individuals, such RAT agents may be considered generally effective and not restricted to only benefiting certain subgroups. This result correlates with AAT literature in which the generic benefits of AAT are promoted as applicable to a range of patients, even when taking individual differences into account.

Co-opting theories from social-cognitive psychology here facilitated the design of a clear, theoretically grounded experiment based on existing research on human–other agent interactions. Instead of simply asking ‘Is PARO a good robot?’, a theoretically grounded exploration of a potential mechanism at work was undertaken, such that it could instead be asked, ‘If this robot is having a positive impact on its user, what mechanism of effect might be occurring here through which that action is happening?’ This was achieved by understanding how humans interact with agents comparably similar to the specific robot here being studied.

Thus, although the results presented in this proof-of-concept work were not wholly conclusive, this opinion paper demonstrates how useful objective, psychological measures of social cognition can be for deepening our understanding of HRI, and developing richer, more robust, HRI methodologies.

## Data accessibility

This article has no additional data.
